# Behavioral barriers to the use of modern methods of contraception among unmarried youth and adolescents in eastern Senegal: a qualitative study

**DOI:** 10.1186/s12889-020-09131-4

**Published:** 2020-06-29

**Authors:** Nicki Cohen, Finou Thérèse Mendy, Jennifer Wesson, Amanda Protti, Carol Cissé, Elhadji Babacar Gueye, Lydia Trupe, Rosii Floreak, Dana Guichon, Karina Lorenzana, Alison Buttenheim

**Affiliations:** 1 ideas42, New York, USA; 2 IntraHealth International, Chapel Hill, USA; 3 University of Pennsylvania School of Nursing, Philadelphia, USA

**Keywords:** Youth, Adolescent, Sexual and reproductive health, Modern methods of contraception, Family planning, Senegal, Sub-Saharan Africa, Barriers, Behavioral diagnosis

## Abstract

**Background:**

Many unmarried young people in low- and middle-income countries (LMIC) want to avoid pregnancy but do not use modern methods of contraception—as a result, half of teen births in these countries are unintended. Researchers have identified numerous barriers that prevent youth from using contraception. However, much of the research in West Africa is narrowly focused on married women, and relatively little research has been done to understand the needs, preferences, barriers, and solution set for sexually active unmarried young people who would like to avoid pregnancy. The purpose of this study was to gain insight into the behavioral barriers that prevent unmarried young people in eastern Senegal from using modern methods of contraception.

**Methods:**

This qualitative study conducted in 2017 in the Tambacounda and Kedougou regions in Senegal explores attitudes and beliefs relating to sex and contraception among unmarried young women and men through 48 in-depth individual interviews with young people aged 15–24 and parents of youth and 5 sex-segregated focus groups with 6–9 young people per group. The research team conducted a thematic content analysis and synthesized the findings by major theme following the behavioral diagnosis methodology.

**Results:**

Drawing insights from behavioral science, the analysis yields five key findings: (1) unmarried young people avoid making a decision about contraception because thinking about contraceptive use provokes uncomfortable associations with a negative identity (i.e., being sexually active before marriage); (2) unmarried young people see modern methods as inappropriate for people like them; (3) unmarried young people are overconfident in their ability to prevent pregnancy through traditional and folk methods; (4) unmarried young people overestimate the social and health risks of modern contraceptive methods; and (5) unmarried young people fail to plan ahead and are not prepared to use modern contraceptive methods before every sexual encounter.

**Conclusions:**

Interventions aimed at increasing uptake of contraceptives among unmarried young people in eastern Senegal must address several significant behavioral barriers in addition to structural, informational, and socio-cultural barriers in order to be successful.

## Background

Access to contraception is imperative for the health and well-being of young people worldwide [1].^1^ Complications from early childbirth are the leading cause of death for adolescent women globally [2]. Half of teen births in low- and middle-income countries (LMIC) are unintended, contributing to four million unsafe teen abortions each year [2]. Unintended pregnancy has significant economic and social consequences such as low levels of education, poor employment opportunities, and inter-generational poverty. Beyond the health, social, and economic consequences, access to sexual and reproductive health (SRH) services has been recognized by the global public health community and intergovernmental organizations as a fundamental human right regardless of marital status, age, parity, or any other individual characteristic [3]; this universal right is enshrined in the U.N. Sustainable Development Goals [4].

Numerous local and international organizations have worked alongside the Ministry of Health and Social Action (MOHSA) in Senegal to improve access to and quality of reproductive health services in recent decades. These initiatives have enjoyed modest success across key indicators, for example increasing the modern contraceptive prevalence rate (MCPR) from 5% in 1993 to 19% in 2017 and reducing the total fertility rate from 6.0 to 4.6 in the same period [5, 6].^2^ However, the vast majority of these initiatives have focused on spacing births among married women, with relatively few resources directed towards unmarried youth [7]. Prevailing social norms in Senegal prohibit sexual activity among unmarried youth, constraining programs directed at this vulnerable group as well as measurement and tracking of their needs and behaviors. For example, Senegal’s Family Planning 2020 (FP 2020) goals set an explicit target for MCPR among married women, but do not mention MCPR for young or sexually active unmarried women [8]. Despite high rates of sexual activity among youth—three-quarters of women who are currently age 25–29 report having had sex before age 24, and half before age 20—MCPR remains low at only 3% for 15–19-year-olds and 14% for 20–24-year-olds [5]. Among sexually active unmarried youth, MCPR is higher (24% among 15–19-year-olds and 48% among 20–24-year-olds), but still leaves significant room for unmet need [5].^3^

Though few studies have investigated the unique barriers to access SRH information and services faced by youth in Senegal, the data available suggest that young people face myriad structural, socio-cultural, and informational barriers. Structural barriers include long distances to the nearest health facility, a lack of trained personnel and supplies, the cost of travel and services, and de facto policies that exclude unmarried young people from SRH information and services [9–13]. Prime among socio-cultural barriers young people face in accessing contraception is the stigma associated with sexual activity, especially among young women [11, 12]. Young people also face informational barriers to using contraception such as a lack of knowledge about the reproductive system, contraceptive options, and the cost and legality of use [11].

While significant research has been done to understand barriers to contraceptive use among married women with children who would like to space or limit future births, relatively little research has been done to understand the needs, preferences, barriers, and solution set for sexually active unmarried young people in Senegal who would like to avoid pregnancy [7]. The purpose of this study was to gain insight into the behavioral barriers that prevent unmarried young people in the Kedougou and Tambacounda regions of eastern Senegal from using modern methods of contraception. In contrast to standard economic models of behavior which assume that people always carefully weigh the benefits and disadvantages of each decision and action, the behavioral approach examines the ways in which cognitive biases and heuristics interact with features of the environment to impact our choices and actions [14, 15]. Results from this study will help reproductive health program designers and policy makers create more effective programs and policies for young people in Senegal.

## Methods

### Study design

We conducted a grounded theory qualitative study based in behavioral science principles using in-depth interviews and focus groups with unmarried young women and men aged 15–24 as well as parents of youth aged 10–24 in the Kedougou and Tambacounda regions of Senegal (see Additional file 1). Data used in this study were collected as part of a foundational research initiative under the United States Agency for International Development (USAID) *Neema* project with the ultimate aim of designing and testing an intervention to reduce unintended pregnancies among unmarried young people in these regions [16].^4^

### Study setting

Our study focuses on two regions in eastern Senegal, Tambacounda and Kedougou, which have particularly high rates of childbearing among young women and a low MCPR. Among 15–19-year-olds in Tambacounda and Kedougou, 30 and 39% have begun childbearing, respectively, about double the national childbearing rate among 15–19-year-olds (16%) [5]. This figure rapidly rises once women reach age 20: among women who are currently 25–29 years old, 67% gave birth before the age of 25 [5].^5^ High rates of childbearing are accompanied by low MCPR in Tambacounda and Kedougou, where fewer than 15% of married women use a modern method, much lower than the national MCPR of 26% [5].^6^ Nationally, among young women who are not currently using contraception, only 5% of 15–19-year-olds and 17% of 20–24-year-olds have spoken with a health worker about family planning in the last year—despite the fact that 44% of 15–19-year-olds and 65% of 20–24-year-olds have visited a health facility or been visited by a community health worker in the last year [5].

### Participant selection

Unmarried youth aged 15–24 were eligible to participate and parents were eligible if they had a child aged 10–24.^7^ Recruitment was carried out by a mixed-gender team of eight Senegalese qualitative researchers in conjunction with community health representatives via purposive sampling in health facilities and the surrounding villages. In villages, the research team walked door-to-door screening all who appeared to be within the relevant age groups and who were receptive to initial questions about interest and availability to participate. In health facilities, the research team conducted similar screening with people who appeared healthy in the waiting area and surrounding streets. As part of the consent procedure, the research team shared that the goal of the study was to learn about participants’ experiences with and attitudes towards health in order to improve reproductive health services for young people. The researchers also mentioned the name and mission of the core organizations associated with the study, the eligibility criteria, and that their answers would remain anonymous, among other consent best practices (see Additional file 4). The recruitment process was identical for individual and focus group participants. Operational considerations (e.g., having a private space available) were the only factor affecting whether young participants were interviewed as individuals or as part of a focus group. Participants were recruited in two health districts from each region: Kedougou and Saraya districts in Kedougou and Koumpentuoum and Kidira districts in Tambacounda. Districts, health facilities, and villages were selected in partnership with regional health administrators in order to maximize the demographic diversity of the populations. No personal information in the form of names or other identifying data was obtained.

### Data collection

We conducted 5 focus groups segregated by gender with 6–9 young people per group and individual interviews with 26 unmarried young people and 22 parents in October–November 2017 (see Table 1).^8^ For both interviews and focus groups, we used open-ended, semi-structured interview guides to explore knowledge, attitudes, and beliefs about sex and contraception over a 45–60-min period (see Additional file 2). Interviews were conducted out of earshot of other community or family members in participants’ homes and public spaces in the village and health facility, depending on availability, by a pair of interviewers with one leading the conversation and another taking thorough notes. Interviews and focus groups were conducted by multilingual researchers in French, Pulaar, Wolof, and Mandinka per the preference of the interviewee. Interviewers and participants were matched by gender when possible, but this was often not possible due to language constraints. Focus group participants were encouraged to share their honest opinions and were asked to verbally commit to keep other participants’ comments private before the session began (see Additional file 2).

**Table 1:** Summary of fieldwork activities conducted in October–November 2017 in Tambacounda and Kedougou, Senegal

	Region (Health Districts)	
	Kedougou (Kedougou and Saraya)	Tambacounda (Koumpentoum and Kidira)
Health facilities visited	• 2 health centers • 3 health posts	• 2 health centers • 4 health posts
Interviews with young people	• 11 unmarried (6 women, 5 men)	• 15 unmarried (10 women, 5 men)
Focus groups with young people	• 2 groups of unmarried men (8–9 men per group) • 2 groups of unmarried women (8–9 women per group)	• 1 group of unmarried women (6 women)
Interviews with parents	• 10 parents (4 mothers, 6 fathers)	• 12 parents (7 mothers, 5 fathers)

### Data analysis

All interviews were audio-recorded with consent. Interview notes were translated to French by the qualitative researchers and then into English by a bilingual member of the research team. The research team conducted a thematic content analysis and synthesized the findings by major theme following the behavioral diagnosis methodology [14]. Prior to the fieldwork, the research team generated a set of hypotheses about potential behavioral barriers to the use of contraception via behavioral mapping, a process that draws insights from the behavioral science literature as well as prior foundational research on the problem and region of focus (see Additional file 3) [17]. After the fieldwork, two members of the research team coded the notes from each interview against the hypotheses in Microsoft Excel in order to identify the hypotheses best supported by the evidence, then synthesized the findings into the results presented in this report. A random sample of interviews equivalent to about 10% of the total number of interviews was listened to and transcribed by members of our research team, confirming that the level of detail and content captured by research team members’ notes matched what was said in the interview.^9^

As a concrete example of the behavioral diagnosis methodology, consider the hypothesis that family planning is seen as exclusively appropriate for married women with families. The research team collected evidence for this hypothesis by asking questions like, “Who is most likely to use family planning?”, “What types of things might prevent young people from going to a health facility to receive family planning?”, and so on. The researchers reviewed the notes from each interview in order to identify and synthesize evidence that supports or refutes this hypothesis in a single spreadsheet. After independently evaluating whether the hypothesis is supported by the synthesized evidence, the team discussed each hypothesis in order to arrive at the results presented here.

## Results

We identified five primary behavioral barriers to the use of contraception among unmarried young people as described below.

Result 1: Unmarried young people avoid making a decision about contraceptive use because thinking about contraceptive use provokes uncomfortable associations with a negative identity.

Parents and most of the youth we interviewed promote abstinence as the optimal behavior for unmarried youth. Contraceptives are seen as a last resort option used only by youth who are promiscuous, lacking in discipline, or unfaithful to their partners. While some young people actively decide not to use contraception, others end up having unprotected sex simply because they have deferred making a decision until it is too late.

*“In our community, young girls who use family planning when they are not married are seen as prostitutes.”*

--Unmarried 18–24-year-old man, Tambacounda.

*“Young girls must abstain until marriage...[they] must not use family planning.”*

--Unmarried 18–24-year-old woman, Kedougou.

*“A woman who is not married shouldn’t need family planning. She must wait until being married.”*

--Mother of 6 children, Tambacounda.

Result #2: Unmarried young people see modern methods of contraception as inappropriate for people like them.

Modern methods of contraception are universally described by the youth and parents we interviewed as most appropriate—or exclusively appropriate—for married women with children who would like to space their births. Complicating matters, modern non-barrier methods of contraception are commonly referred to as “family planning”, underlining that their intended users are married couples planning their families. Even when youth do think about their options for avoiding pregnancy, modern methods other than condoms are not in their choice set.

*“If it’s the case of an unmarried young woman, they [the community] will think that she uses family planning so she can prostitute herself. [For a married woman], they think that she has done a good thing.”*

–Unmarried 18–24-year-old woman, Kedougou.

*“Only married young women use family planning. In our community, young unmarried women don’t use it. For boys, they will get condoms.”*

--Unmarried 18–24-year-old woman, Tambacounda.

*“If I found my unmarried daughter were using family planning, I would deprive her of everything.”*

--Father of 5 children, Tambacounda.

Result 3: Unmarried young people are overconfident in their ability to prevent pregnancy through traditional methods of contraception.

Youth undervalue modern contraceptive methods because they believe they are adequately protected from pregnancy by traditional methods (such as withdrawal) or folk methods (such as amulets).^10^ Many young people that we spoke with do not see modern methods as a more effective replacement for traditional and folk methods, but rather as an equal alternative. Compounding this issue, many youth hold incorrect beliefs about the menstrual cycle that further undercut the effectiveness of the rhythm method, the traditional method that youth mention most often.

*“They [unmarried young people] can go to health facilities to use family planning or go see marabouts. Traditional methods are also reliable.”*^10^

--Father of 9 children, Tambacounda.

*“The girl could use family planning or the boy could use condoms. Or they could abstain during the period that it’s most likely that she will become pregnant. There are also those who use traditional talismans as a method of family planning.”*

--Unmarried 18–24-year-old woman, Tambacounda.

*“If she is knowledgeable about it [her cycle], she doesn’t have to use family planning. After her period, she should wait 5 days to have sex, and nothing will happen.”*

--Young male focus group participant, Kedougou.^11^

Result #4: Unmarried young people overestimate the health and social risks associated with modern methods of contraception.

The youth we interviewed significantly overestimate the health risks of modern methods of contraception. Negative anecdotes about health issues stemming from the use of modern methods—especially hormonal methods—are widely circulated and seen as credible.

*“It’s dangerous because family planning can disappear in your body and you can no longer have children.”*

--Young female focus group participant, Tambacounda.

“When a woman uses family planning before marriage, she risks becoming infertile.”

--Unmarried 15–17-year-old man, Tambacounda.

Youth also overestimate the social stigma associated with using contraception. While many young people we spoke with think that community members would unconditionally disapprove of their use of contraception, interviews with parents suggest a slightly more nuanced story. Though none of the parents we interviewed fully endorsed the use of contraception among their unmarried children, many said they would support it if their child were at a high risk for pregnancy.

*“I would be ashamed to get condoms at the health hut because my uncle is there. When we go to get condoms, we don’t want to meet people we know at the hospital.”*

–Young male focus group participant, Kedougou.

*“The proximity of the facility if they are not married [could prevent young people from getting contraception]. Because people will say that the daughter of so-and-so used family planning even though she isn’t married so she had sex outside of marriage.”*

--Unmarried 15–17-year-old woman, Tambacounda.

*“If my child ... became pregnant, I would be ashamed. So if my child personally wants to use family planning to avoid getting pregnant, I would accept that.”*

–Father of 12 children, Kedougou.

Result 5: Unmarried young people fail to plan ahead and are not prepared to use modern contraceptive methods before every sexual encounter.

In Kedougou and Tambacounda, discussion of sex and contraceptives is considered taboo. Only a few of the young people we interviewed reported discussing contraceptive use with their partner ahead of sex, instead preferring to discuss sexual health topics with friends of their own gender. A further complicating factor is that while the most common form of contraception for unmarried people (the male condom) is seen as the responsibility of young men, young women bear the majority of the social consequences for a pregnancy outside of marriage. Women who become pregnant are stigmatized within the community—they may be thrown out of their parents’ house, forced to leave school, or beaten—while young men may be able to deny their responsibility for the pregnancy and escape the social and financial consequences of caring for the child.^12^ Because men can decline their responsibility for the pregnancy, ensuring they use protection with every sexual encounter may not feel like a high priority.

*“It’s nothing but misery [if you become pregnant outside of marriage]! They beat you, they insult you, you don’t eat, people talk about you...People call you a degenerate.”*

--Unmarried 18–24-year-old woman, Tambacounda.

*“If a boy discovers that you are pregnant then you are screwed because even if it is the boy who got you pregnant, he will deny it because if you had sex with me, then you could be having sex with someone else.”*

–Young female focus group participant, Tambacounda.

Unmarried young people may intend to use contraceptives every time they have sex, but if they do not have condoms on hand or expect condoms to reduce their physical pleasure, they may ignore their intention to use a condom due to visceral feelings of arousal.

*“During my last time having sex, I didn’t use a condom. I looked for one, but I couldn’t find one.”*

–Unmarried 18–24-year-old man, Kedougou.

*“When I talk to them about sex, some of my friends are so immature. They say, ‘Does rice have the same taste everywhere? All rice has its own flavor. And if we protect ourselves with a condom, we will not have the desired taste.’”*

--Unmarried 18–24-year-old man, Tambacounda.

## Discussion

Unmarried youth in eastern Senegal face many of the same barriers that married adults in West Africa and other LMIC face in order to access modern methods, such as fears of infertility and stigma associated with contraceptive use [18]. However, we also identified behavioral barriers unique to our study population that programs and policies targeting unmarried youth in eastern Senegal will need to confront, such as young people’s overconfidence in the effectiveness of traditional and folk methods and overestimation of the social and health risks of using contraception. Each barrier is facilitated or exacerbated by cognitive biases, leading youth to avoid making a decision about contraception, decide not to use contraception, or fail to follow through on an intention to use contraception during every sexual interaction, as described below.

Two cognitive biases that contribute to youth’s avoidance of contraceptive decisions are *cognitive dissonance* and incorrect *mental models*. In a society that views premarital sex as categorically negative, thinking about contraception causes cognitive dissonance—a mental discomfort with any action that contrasts with one’s identity, values, or beliefs—leading youth to avoid seeking information about or making a decision to use contraception [19, 20]. On top of this, youth we interviewed hold the mental model that family planning is primarily or exclusively for married women to space births, reflecting Senegalese society’s widely held views on contraception [7, 12]. Mental models are simplified cognitive representations based on prior knowledge and experience that we use to understand and predict complex mechanisms in the world around us and that, as a result, often guide our behavior. The mental model that family planning is not meant for youth reduces the likelihood that youth will even consider it [21].

Youth who actively consider contraception may decide not to use it due to erroneous estimations of the risks and benefits of doing so which are likely to be impacted by incorrect mental models, *overconfidence*, *the availability heuristic*, and *present bias*. The incorrect belief that traditional and folk methods are as effective as modern methods lowers the relative value of highly effective modern methods [22, 23]. Reliance on traditional methods is problematic for two reasons: 1) with common usage they are far less effective than modern methods, and 2) given young people’s lack of knowledge about the menstrual cycle, the traditional method mentioned most frequently (rhythm method) is unlikely to be applied correctly.^13^ In the most recent national household survey, only 58% of current users of the rhythm method could correctly identify the fertile period, and only 11% of 15–19-year-old-women and 22% of 20–24-year-old women could do so [5].

Rather than relying on traditional or folk methods, some youth may believe they do not need modern methods of contraception simply because they do not intend to have sex. Striving to meet the community norm of abstinence and maintain a positive sense of self, youth in Senegal tend to be overconfident in their ability to abstain, leading them to underestimate their risk of pregnancy and the value they might derive from using contraception [24]. Overconfidence is a universal constant—74% of motorists think they are better at driving than the average driver, for example [25, 26]. Overconfidence in self-control is particularly detrimental as it likely leads young people to reduce efforts to avoid tempting situations [27].

On the risk side, widespread rumors about the negative health impact of modern methods cause the youth we interviewed to overestimate the health risks (particularly risk of infertility) due to the availability heuristic, a mental shortcut we use to evaluate risks in which we perceive risks that come to mind easily as more likely to occur [28, 29]. Senegalese society places a high value on fertility. Across sub-Saharan Africa, women who are not able to bear children are significantly devalued and face negative psycho-social outcomes from social rejection and isolation to divorce and physical abuse [30, 31]. For a young woman, any risk that would endanger her future fertility, however small, is to be avoided.

Another bias that likely contributes to youth’s overestimation of the risks and underestimation of the benefits of modern methods is present bias. Because present bias causes near-term benefits and costs to feel bigger or more important than those in the longer term, the social stigma and embarrassment youth expect to experience when visiting a health facility or pharmacy to access contraceptives are amplified compared to the longer-term, uncertain, and rather abstract benefit of remaining free of pregnancy. The potential future social stigma, health risks, and financial costs from an unintended pregnancy are likely to be underweighted in current decisions [32].

Even youth who intend to use contraception may not do so with every sexual encounter due to *diffusion of responsibility* and *the hot-cold empathy gap*. In cases where there is no conversation about contraceptives ahead of a sexual encounter, neither partner feels like they have the responsibility for preventing pregnancy. This diffusion of responsibility has been shown to inhibit action across a range of contexts: people work less hard, are less likely to help others, and are more likely to take risks when others are present and jointly able to take responsibility [33].

Lastly, young people who intend to use contraceptives may not always do so because of the hot-cold empathy gap. When people are in a “cold” state—calm, rational, and unemotional— they are unable to accurately anticipate how they will feel when they are in a visceral, or “hot,” state— angry, hungry, or in pain [34]. Sexual arousal is a characteristic example of a “hot” state, and has been shown to reduce intentions to use protection during sex [35].

### Programmatic implications

Standard interventions targeting youth uptake of SRH services—such as youth-friendly service delivery training for providers, youth centers, and peer educators—have failed to reliably improve SRH outcomes for youth [36, 37]. Multi-faceted approaches that combine health worker training, adolescent-friendly facility improvements, and information dissemination via the community, schools, or media have seen positive but weak results [37]. We believe these standard interventions fail to significantly shift youth behavior in part because they are designed with the assumption that providing information and access to contraception is enough to change behavior. Key to the behavioral science approach is the firm belief that information and access are necessary but not sufficient. Interventions must address behavioral barriers in addition to informational and structural barriers in order to be successful.

Additionally, standard interventions to increase youth uptake of SRH services are time and resource intensive [36]. Because behavioral approaches often build on existing infrastructure and programs, they can be highly cost-effective. Though they may be limited in scope by existing channels to reach youth, behavioral elements such as re-framing a text or voice message, providing a micro-incentive, utilizing checklists, or goal-setting activities often add only marginal costs to existing programs and policies [38].

One promising way forward is to incorporate behavioral elements into future interventions in order to help young people form an intention to use contraception and then follow through on that intention. An effective approach might begin by creating a moment of choice for young people to thoughtfully consider their pregnancy risk and whether contraceptives might be beneficial for them. In order to counteract the behavioral barriers described in this report, it would be critical in this moment to highlight positive identities of a young contraceptive user (e.g., responsible for future, choosing when to have a baby in order to have a healthier baby), reframe the norm about intended users of modern methods to include unmarried women and youth, and dispel common myths about the side effects of modern methods. For youth who have decided to use contraception, the design should include concrete and actionable plan-making tools and timely reminders in order to help youth follow through on their intentions.

For example, our research team recently designed a novel intervention informed by the findings in this report in which youth are given appointments for a free wellness checkup with the local nurse or midwife. The checkup includes several low-stigma themes relevant for youth (such as exercise and nutrition) in addition to carefully framed reproductive health questions and messages that aim to bust myths about contraception and help youth make a thoughtful, well-informed contraceptive choice. Results from a feasibility pilot in five health facilities in Tambacounda with over 200 youth indicate that the design is feasible to implement, increases youth visits to health facilities, and has the potential to shift youth contraceptive behavior.

### Limitations

Findings from the study are not representative of all districts in Tambacounda and Kedougou. The majority of interviews were not fully transcribed, and some details may not have been fully or correctly captured in researchers’ notes. Our interview guides were developed with the aim of uncovering behavioral barriers to contraceptive use. This paper does not examine structural, social, and informational barriers to contraceptive use. The findings are also subject to general limitations on qualitative research in terms of validity, reliability, and subjectivity [39].

## Conclusions

Standard interventions to increase access to SRH services among youth are not working, and without significant innovation Senegal and other LMIC are unlikely to hit their FP2020 or 2030 Sustainable Development Goals. A major reason why these interventions fail to significantly shift outcomes is that they overlook the behavioral barriers preventing youth from deciding to use contraception and following through on that intention. Identifying the cognitive biases and features of their environmental that underlie these barriers is the first step towards addressing them in effective programs and policies.

We recommend that future programs and policies aiming to increase contraceptive use among youth in eastern Senegal or similar populations address the behavioral barriers highlighted in this paper in addition to structural and informational barriers. In order to be successful, such interventions must facilitate an informed and deliberate decision about contraceptive needs, lower the perceived health and social risks of accessing reproductive health information and services, highlight the benefits of modern methods over traditional and folk methods, and aid follow-through on intentions to access and use contraception.

Finally, we recommend that the Ministry of Health and Social Action in Senegal revise their FP2020 and other future family planning goals to include specific targets for unmarried youth – after all, ‘what gets measured gets managed’ ([40]: p.2]).

## Supplementary information

**Additional file 1.** Completed COREQ checklist.

**Additional file 2.** Blank English interview guides.

**Additional file 3.** Illustrative behavioral hypotheses.

**Additional file 4.** Consent forms.

## Data Availability

The datasets generated and analyzed during the current study are not publicly available in order to protect the privacy of the participants but are available from the corresponding author on reasonable request.

## Abbreviations

FP2020: Family Planning 2020
LMIC: Low- and middle-income countries
MCPR: Modern contraceptive prevalence rate
MOHSA: Ministry of Health and Social Action
SRH: Sexual and reproductive health
USAID: United States Agency for International Development

## Footnotes

1 We use the UN’s definition of youth, which includes people aged 15–24.

2 Modern methods available in Senegal include male and female sterilization, injectables, IUD, oral contraceptives, implants, male and female condoms, Standard Days Method, lactational amenorrhea method, and emergency contraception.

3 Unmet need is 38% for unmarried sexually active women of all ages, but is not reported for unmarried sexually active youth.

4 The goal of the Neema project is to improve health for women and children in Senegal by strengthening health services and making them accessible to more people, including increasing access to and use of modern contraception in public health facilities and communities, encouraging youth-friendly healthcare, and promoting behavior change by providing the knowledge and resources needed to make informed, healthful decisions.

5 Note figure represented on national level as figures for Tambacounda and Kedougou are not available.

6 Regional MCPR is not reported for unmarried women or by age group.

7 We also conducted individual in-depth interviews with 2 midwives and 7 married youth aged 15–24; however, these findings have been excluded from the analysis.

8 We did not track the number of people who refused to participate. Two unmarried young women and one father were dropped from the analysis due to incomplete interviews. Prior formative research conducted in Tambacounda and Kedougou in February–March 2017 included one-on-one conversations with 10 young people (five men and five women) and 14 health workers (ten midwives, three head nurses, and one laboratory technician); group conversations with six groups of young people (three groups of four-eight women and three groups of four-eight men); and observations at nine health facilities (four health centers, four health posts, and one youth health center).

9 Six sets of notes were identified as unreliable due to duplication across three pairs of notes. These six sets of notes were fully transcribed from the audio recording (in addition to the 10% sample). Transcriptions replaced interviewer notes in our evidence base when available.

10 Traditional contraceptive methods include the rhythm (or calendar) method and withdrawal. Folk methods such as amulets or herbal remedies are provided by traditional healers called marabouts.

11 We did not collect exact ages for all focus group participants.

12 Abortion is illegal in Senegal except as a last resort in order to save the mother’s life; significant bureaucratic hurdles prevent access to safe and legal abortion even in these extreme cases [11].

13 The misperceptions of effectiveness described in this study apply only to the rhythm method and not to fertility awareness methods that are considered to be modern methods.
