# Supplementary material for: Behavioral barriers to the use of modern methods of contraception among unmarried youth and adolescents in eastern Senegal: a qualitative study
Source: BMC Public Health. 2020 Jun 29;20:1025. doi: 10.1186/s12889-020-09131-4 (PMC7325306; doi:10.1186/s12889-020-09131-4)
Supplement: Supplementary file 2 — Additional file 2. Blank English interview guides. [file 12889_2020_9131_MOESM2_ESM.docx]

Interview & focus group guides

**Interview Guide 1: Unmarried Young People At Health Facility**

**Background/Icebreakers**

1. To start, can you tell me a little about yourself?
   1. Who do you live with?
   2. How old are you?
   3. Are you in school?

**Experience with Health Services**

1. What brought you to the health center/post/hut today?
   1. What services did you receive?
   2. How much did you pay for the services you received today? Were there any other costs? (probe: transportation)
2. Can you tell me a little bit about the process when you came to the health facility today?
3. How did you get here and how long did it take? (walk, drive, other transportation) What time did you leave your house? What time did you arrive?
4. How long did you wait to be seen? What time were you seen?
5. Did you come alone or with someone else? Who?
6. Why did you choose to come at this time? Did you have to interrupt any other activities (work, childcare, school, housework) to come?
7. Did anyone know you were going to a health facility today? How did they know? (did you tell them/did they see you coming)
8. Who did you interact with when you came today? What was that person’s (or people’s) role? (Probe: midwife, head nurse, community health worker, or someone else?)
9. What were those interactions like?
   1. Did the health worker give you a chance to ask any questions? Did you have any questions for health workers?
   2. Did anything make you uncomfortable while you were talking to the health workers? (probe: confidentiality, privacy, bias)
   3. What did you think of the service provided? Do the staff meet your needs? (Did you receive all of the services/treatment you came for?)
10. Which health worker do you prefer to talk to about things that are very private? Why?
11. Is there any other place you go to if you are sick or have a health issue? (probe: traditional medicine, family/friends, pharmacy) Why do you go there?
12. For which issues?
13. What services do you receive?

**Reproductive Health Knowledge**

1. Have you ever received any information about family planning (methods to prevent a pregnancy) ?
   1. On what occasion did you receive this information? (Probe: school, care group, community outreach, health facility, media)
2. Who would you ask if you wanted to learn more or if you had a question about family planning? (probe: school, mother, sister, aunt, friends)
   1. Why would you ask this person rather than someone else?
3. Do you ever talk with your friends about sex? What kinds of things do you talk about? (probe: sexual intercourse, illness, FP, unwanted pregnancy)
   1. Why did you decide to talk to your friends about these issues? (probe: solution to a problem, acting as a *relais*)

**Reproductive Health Norms, Attitudes, and Behaviors**

1. Now I want to talk a little bit about people your age in this community. How common is it for people your age (refer to specific age: high school age, early 20s, etc.) to be having sex?
   1. At what age do young men in this community usually start having sex? What about young women?
2. How do you know that someone is having sex?
3. How likely is a woman to get pregnant if she has unprotected sex?
4. Are unmarried young people often in relationships? (probe: age/gender differences)
5. What would happen if an unmarried woman becomes pregnant?
6. What do people think of unmarried women who become pregnant?
7. If someone your age is having sex but does not wish to become pregnant, what do they do?
8. What options/methods have you heard of for preventing pregnancy?
9. Do people in this community who are your age ever use family planning (or any method of preventing pregnancy)?
10. What benefits have you heard of for using family planning? Negatives?
11. Do you know where you could get family planning methods? (probe: health center, health post, a pharmacy/dispensary, a community health worker, friends)
    1. Would you have to pay? If yes, do you know how much?
12. Who is most likely to use family planning? (probe: age, gender, marital status)
13. Who do you think should use family planning? (probe: age, gender, marital status)
14. What do people think of those who are using family planning? (probe: positives and negatives, age/gender/marital status differences)

**“Now I want to ask a little bit about your personal experiences with intimate relationships.”**

1. Have you ever been in a relationship (for example having a boy/girlfriend or a husband/wife)? (probe: single, couple, marriage, length of relationship)
   1. IF YES:
      1. Did you and your partner ever engage in sexual activity?
   2. IF NO:
      1. Have you had any sexual partners over the past year?
   3. IF YES TO ANY SEXUAL ACTIVITY:
      1. Were you nervous about getting pregnant/her getting pregnant? Why or why not?
2. Have you ever used any family planning methods before (methods to prevent pregnancy)?
   1. Which type of method(s) have you used?
   2. Where did you receive/purchase the family planning method?
   3. How did you decide which method to use? Did anyone help you make this decision?
   4. Did anyone else know you were using a family planning method?
   5. What was the process for taking up family planning? Describe the steps.
   6. How did the health worker respond when you asked to use family planning?
   7. How much did the FP method cost?
   8. Did you have a positive or a negative experience using FP? What were the positives/negatives?
   9. Are you still using a family planning method?
   10. Did you use a family planning method the last time you had sex? If so, which method? (probe: condoms)
3. What types of things might prevent young people like you from going to a health facility to receive family planning? (probe: cost, quality, hassles)
4. If you were looking for a place to receive family planning, what factors would be important for you when choosing a health facility?
5. Is there anything else that you’d like to share about family planning or youth health services?

**Conclusion:** We are at the end of the interview. Do you have any questions or comments? Thank you for taking time to talk to us.

**Interview Guide 2: Young People (In the Community)**

**Background/Icebreakers**

1. To start, can you tell me a little about yourself?
   1. Who do you live with?
   2. How old are you?
   3. Are you in school?

**Experience with Health Services**

1. Have you ever visited a health facility (health center/hospital/health post/health hut)?
   1. IF YES:
      1. When is the last time you went to a health facility and why?
      2. How much did the services cost? Are there any other costs? (probe: transportation)
   2. IF NO:
      1. Go to question #9
2. Can you tell me a little bit about the process when you use a health facility?
3. How do you get there and how long does it take? (walk, drive, other transportation)
4. What is the waiting time like?
5. Do you go alone or with someone else?
6. How often do you go to health facilities?
7. What time do you usually go to health facilities?
8. How convenient is it to go to the health facility?
9. During your last health visit, did anyone know you were going to the health facility? How did they know? (did you tell them/did they see you coming)
10. During your last health visit, whom did you interact with? What was that person’s (or people’s) role? (probe: midwife, head nurse, community health worker, or someone else)
11. What were those interactions like?
    1. Did the health worker give you a chance to ask any questions? Did you have any questions for health workers?
    2. Did anything make you uncomfortable while you were talking to the health workers? (probe: confidentiality, privacy, bias)
    3. What did you think of the service provided? Did the staff meet your needs? (Did you receive all of the services/treatment you came for?)
12. Which health worker do you prefer to talk to about things that are very private? Why?
13. Is there any other place you go to if you are sick or have a health issue? (probe: traditional medicine, family/friends, pharmacy) Why do you go there?
14. For which issues?
15. What services do you receive?
16. If youth has no experience with a health facility:
17. What services do they offer at health facilities? Which people go to health facilities? Do you know other people your age who have been?
18. Where do you receive treatment for illness?
19. If you have a question about your health, who do you ask?

**Reproductive Health Knowledge**

1. Have you ever received any information about family planning?
   1. On what occasion did you receive this information? (Probe: school, care group, community outreach, health facility, media)
2. Who would you ask if you wanted to learn more or if you had a question about family planning? (probe: school, mother, sister, aunt, friends)
   1. Why would you ask this person rather than someone else?
3. Do you ever talk with your friends about sex? What kinds of things do you talk about? (probe: sexual intercourse, illness, FP, unwanted pregnancy)
   1. Why did you decide to talk to your friends about these issues? (probe: solution to a problem, acting as a *relais*)

**Reproductive Health Norms, Attitudes, and Behaviors**

1. Now I want to talk a little bit about people your age in this community. How common is it for people your age (refer to specific age: high school age, early 20s, etc.) to be having sex?
2. At what age do young men in this community usually start having sex? What about young women?
3. How do you know that someone is having sex?
4. How likely is a woman to get pregnant if she has unprotected sex?
5. Are unmarried young people often in relationships? (probe: age/gender differences)
6. What would happen if an unmarried woman becomes pregnant?
7. What do people think of unmarried women who become pregnant?
8. If someone your age is having sex but does not wish to become pregnant, what do they do?
9. What options/methods have you heard of for preventing pregnancy?
10. Do people in this community who are your age ever use family planning (or any method of preventing pregnancy)?
11. What benefits have you heard of for using family planning? Negitives?
12. Do you know where you could get family planning methods? (probe: health center, health post, a pharmacy/dispensary, a community health worker, friends)
    1. Would you have to pay? If yes, do you know how much?
13. Who is most likely to use family planning? (probe: age, gender, marital status)
14. Who do you think should use family planning? (probe: age, gender, marital status)
15. What do people think of those who are using family planning? (probe: positives and negatives, age/gender/marital status differences)

“Now I want to ask a little bit about your personal experiences with intimate relationships.”

1. Have you ever been in a relationship (for example having a boy/girlfriend or a husband/wife)? (probe: single, couple, marriage, length of relationship)
   1. IF YES:
      1. Did you and your partner ever engage in sexual activity?
   2. IF NO:
      1. Have you had any sexual partners over the past year?
   3. IF YES TO ANY SEXUAL ACTIVITY:
      1. Were you nervous about getting pregnant/her getting pregnant? Why or why not?
2. Have you ever used any family planning methods before (methods to prevent pregnancy)?
   1. Which type of method(s) have you used?
   2. Where did you receive/purchase the family planning method?
   3. How did you decide which method to use? Did anyone help you make this decision?
   4. Did anyone else know you were using a family planning method?
   5. What was the process for taking up family planning? Describe the steps.
   6. How did the health worker respond when you asked to use family planning?
   7. How much did the FP method cost?
   8. Did you have a positive or a negative experience using FP? What were the positives/negatives?
   9. Are you still using a family planning method?
   10. Did you use a family planning method the last time you had sex? If so, which method? (probe: condoms)
3. What types of things might prevent young people like you from going to a health facility to receive family planning? (probe: cost, quality, hassles)
4. If you were looking for a place to receive family planning, what factors would be important for you when choosing a health facility?
5. Is there anything else that you’d like to share about family planning or youth health services?

**Conclusion:** We are at the end of the interview. Do you have any questions or comments? Thank you for taking time to talk to us.

**Interview Guide 3: Parents**

**Background/Icebreakers**

1. To start, can you tell me a little about yourself and your family?
2. Are you married?
3. Do you have children? How many? What are their ages?
4. Are any of your children married? Do they have children?

**Experience with Health Services**

1. How often do you visit health facilities (health center/hospital/health post/health hut)? For which services?
2. Do you go alone or with someone else?
3. How often do young people (age 15-24) go to health facilities?
4. For what services do young people go to health facilities?
5. What are some reasons why young people might not want to go to a health facility?
   1. What are some things that would make young people **more** likely to go to a health facility?

**Reproductive Health Knowledge**

“Next I want to talk about some health issues relating to intimate relationships between young women and men”

1. What do you think young people in this community know about sex and relationships?
2. How do they learn about these topics? (probe: school, friends, family, community sensitization)
3. What unanswered questions / worries do you think your child (age 15-24) has about these topics?
4. Have you ever talked to your child (age 15-24) about sex and relationships?

[IF NO TO 10, ASK QUESTION 11]

1. If no, why not? How comfortable would you be to talk to your child about these topics if they asked?

[IF YES TO 10, ASK QUESTIONS 12-15]

1. What did you talk about? (probe: sex, family planning)
2. What kinds of questions did your child ask?
3. Who initiated this conversation (you or your child)? What prompted the conversation?
4. How did this conversation make you feel? How did your child react to the conversation? (Probe: attitude, comfort)

**Reproductive Health Experience**

“Now I want to talk a little bit about intimate relationships between young men and women in this community.”

1. How does a young person decide when to become sexually active? (probe: marriage, relationship, age)
2. How common is it for young people (age 15-24) to be having sex?
   1. At what age is it normal for young men to start having sex? What about young women?
   2. Is it common for young people to have sex when they are not married?
3. How would you know that a young person is having sex?
4. How likely is a young woman to get pregnant if she has unprotected sex?
5. What would happen if an unmarried young woman becomes pregnant?
6. What do people think of unmarried women who become pregnant?
7. If an unmarried young person is having sex but does not wish to become pregnant, what do they do?
   1. What have you heard young people using?
   2. What **should** they do?
8. Who is most likely to use family planning? (probe: age, gender, marital status)
9. Who do you think should use family planning? (probe: age, gender, marital status)
10. Do you know where unmarried young people can get any of these methods? (probe: health center, health post, a pharmacy/dispensary, a community health worker, friends)
11. What do people think of unmarried young people who are using family planning? (probe: positives and negatives, age/gender/marital status differences)
12. Do you know if any of your children have ever used family planning? Which method?

[IF YES TO 27 ASK 28-30]

1. Why did your child decide to use family planning?
2. Did you and your child discuss this before your child went to the health facility? Who initiated this conversation?
3. How do you feel about your child’s use of family planning?

[IF NO TO 27, ASK 31-33]

1. Would you ever want your child to use family planning? Under which circumstances? (i.e. what are some reasons why you would want your child to use these services?)
2. How would you feel if your child wanted to use family planning?
3. Are there any reasons why you would not want them to use these services? What are these reasons?
4. Is there anything else that you’d like to share about family planning or youth health services?

**Conclusion:** We are at the end of the interview. Do you have any questions or comments? Thank you for taking time to talk to us.

**Focus Group Guide —Young People**

“We want everyone to feel comfortable sharing their thoughts with us. Remember, everything that we talk about here today is private. You shouldn’t share anything that someone else in the group says with anyone else in the community. **Does everyone agree to keep this conversation private?”**

**Warm up/Icebreakers**

Ask everyone to say their name and hobby or favorite type of music

**Health Services for Youth**

1. We are here to learn more about health services for youth in this community. Can you describe some of the services people your age might go to a health facility (health center, post, or hut) for?
2. What is the process like for people your age who want to go to a health facility? Is it easy or hard? Why?
3. What criteria do you prioritize when choosing a health facility? (probe: time, provider, quality of services and care, cost, confidentiality)

**Reproductive Health Norms, Attitudes, and Behaviors**

1. Now we want to talk about some of the norms in this community. What types of relationships often exist between young men and women your age? (probe: marital status, boyfriends/girlfriends, single, friendships)
   1. Are unmarried people your age often in relationships? (probe: age/gender differences)
2. Is it common for unmarried people your age (refer to specific age: high school age, early 20s, etc.) to have sex?
   1. At what age do young men in this community start having sex? Young women?
   2. Do people usually wait until they are married to have sex or do they have sex before they are married? (probe: relationship status, gender)
3. What do you think about unmarried people your age having sex?
4. Do people your age (refer to specific age: high school age, early 20s, etc.) ever have sex when they do not want to become pregnant?
   1. If yes, what do they do to prevent pregnancy? (If they talk about family planning, ask about methods)
5. How likely is a woman to get pregnant if she has unprotected sex?
6. What would happen if an unmarried young woman becomes pregnant?

**Family Planning Use and Attitudes**

1. What do people think of young people who are using family planning? (probe: positives and negatives, age/gender/marital status differences)
2. What are some common options (or methods) for family planning among young people in this community?
3. How do young people decide whether to use family planning (and what method to use)?
   1. Do they usually make the decision alone or do other people influence their decision (probe: parents, partner, health workers, friends)?

**Family Planning Services and Information in the Community**

1. Where can young people go to receive family planning services?
2. What are the most important factors that young people consider when deciding whether to go to a health facility for family planning? (probe: distance, types of health workers, hours)
3. How easy or hard is it for young people to use family planning? Why?
4. Why might people young people not go to a health facility for services? (probe: cost, bias, quality of care)
5. Are there other places where young people might prefer to go for these services?
6. Who do young people usually talk to if they want more information about family planning? (probe: parents, teachers, health workers, family, friends, partners, peer educators)
   1. What makes these conversations easy or difficult?
7. Is there anything else that you’d like to share about family planning or youth health services?

**Conclusion:** We are at the end of the discussion. Do you have any questions or comments? Feel free to come talk to me afterwards if there is anything else you would like to discuss. Thank you for taking time to talk to us. Please remember that we all agreed to keep this conversation private.
