# Supplementary material for: Behavioral barriers to the use of modern methods of contraception among unmarried youth and adolescents in eastern Senegal: a qualitative study
Source: BMC Public Health. 2020 Jun 29;20:1025. doi: 10.1186/s12889-020-09131-4 (PMC7325306; doi:10.1186/s12889-020-09131-4)
Supplement: Supplementary file 3 — Additional file 3. Illustrative behavioral hypotheses. [file 12889_2020_9131_MOESM3_ESM.docx]

Illustrative list of hypotheses generated via behavioral mapping that informed the interview and focus group guides

| **Hypothesis** | **Relevant behavioral principle(s)** |
| --- | --- |
| There are few/no external cues to make a decision about whether to use family planning | Salience, limited attention, status quo bias |
| Women underestimate their likelihood of becoming pregnant from unprotected sex | Overconfidence, mental models, base rate neglect |
| Family planning is seen as appropriate only for married women who have families | Identity, mental models, social norms |
| The consequences of not using family planning are far in future, while the costs (both real and perceived) of taking up family planning feel imminent | Present bias, mental models |
| Young people prefer to eliminate risk of any social stigma (by not using contraceptives) vs having some risk of social stigma and a reduced risk of pregnancy (by using contraceptives) | Zero risk bias, mental models |
| Young people do not to take up contraceptives because they are unsure of the risks (and it feels difficult to figure out what they are) | Ambiguity aversion |
| Young people feel that using contraceptives would acknowledge a sexual identity; negative feelings towards unmarried sexual behavior discourage young people from even thinking about taking up family planning | Negative affect, ostriching, identity |
| Youth see pregnancy and contraceptive use as outside of their control (it is God's choice, partner's choice, parents' choice...) | Locus of control, social norms, diffusion of responsibility |
| Young people expect to be scolded or negatively judged by health workers if they request family planning services | Availability bias, social norms, confirmation bias |
| Young people assume they will be socially ostracized for using family planning and are afraid/embarrassed about being seen at the health post | Social norms, spotlight effect |
| Talking to a more educated health worker primes an identity of being uneducated or poor and thus deters young people from seeking services at health facilities | Identity, negative affect |
| Young people do not think about family planning until they are in the moment of sexual activity | Hot-cold empathy gap, overconfidence |
| Finding transportation to the facility and waiting in line are hassles that may not seem worth it, especially if the benefits of family planning are unclear | Hassles, present bias |
