# Supplementary material for: Behavioral barriers to the use of modern methods of contraception among unmarried youth and adolescents in eastern Senegal: a qualitative study
Source: BMC Public Health. 2020 Jun 29;20:1025. doi: 10.1186/s12889-020-09131-4 (PMC7325306; doi:10.1186/s12889-020-09131-4)
Supplement: Supplementary file 4 — Additional file 4. Consent forms. [file 12889_2020_9131_MOESM4_ESM.docx]

Consent forms

**RESEARCH PARTICIPANT CONSENT FORM (YOUNG PEOPLE AGED 18-24 & EMANCIPATED MINORS)**

**Project Title:** Neema: Formative Research on the Use of Reproductive Health Services among Youth in Kédougou and Tambacounda

IRB number: N° 00139/MSAS/DPR/CNERS du 23 Oct 2017

**Introduction:**

Hello, my name is ______________________ and I work for______________________. I am here with IntraHealth International and ideas42, two American organizations whose goal is to improve public health. We are working with the Ministry of Health and Social Action to improve reproductive health services for young people. To do this, we seek to understand the various problems they face in order to remedy them. It is important that we understand the knowledge, attitudes, and behaviors related to their health. The information we get will allow us to make recommendations.

**Why were you chosen?**

You were selected because you are between the ages of 15-24. However, not all young people your age will be able to participate in this study. If you agree to participate in this research, you can help us understand the needs of youth your age in your community. By agreeing to participate, you will be asked to answer questions about your knowledge and opinions about the reproductive health services available to young people, including family planning and testing for HIV and STIs.

**Should I participate?**

Your participation is entirely voluntary. If you agree, you will be asked to sign a consent form. You may stop answering questions at any time for any reason. We are talking with a lot of other people. Anything you say will remain anonymous. We will not share your name or any other personal information. This will take 30 to 40 minutes.

**What are the disadvantages or potential benefits of participating?**

You will not benefit directly from your participation. But your opinion will help us to come up with recommendations to improve the health of young people your age. It is unlikely that there will be any risks or disadvantages in participating in this research. If you are uncomfortable with some questions, you can end your participation on the spot or move on to another question.

**What if I need more information or if I have questions?**

For further information or comments on this research, please contact the Principal Investigator (details below).

**Will my participation in this study be kept confidential?**

All information you give us will remain anonymous. Your name will not appear on any document. All data collected will remain confidential, the information we receive from you will be shared with other individuals for the purposes of the analysis, but in no way will we know who gave this information. We will work to remove any information that could identify you.

**Do you have any questions?**

If you agree to participate in this research, sign below. You will be given a copy of the form you will keep.

**Please check:**

| 1. I confirm that I have received all the information and details for the participants. I have had the opportunity to ask questions and I received a full answer to those questions. |  |
| --- | --- |
| 1. I understand that my participation is voluntary and I am free to withdraw at any time, without giving any reason. |  |
| 1. I agree to participate in the above study. |  |
| 1. I agree to the recording of the interview. |  |
| 1. I agree that the research team can use anonymous quotes from my interview in the final report and in any other product of this research. |  |

**Signature** : ...........................................................Date ........................................................................

**PARENTS / LEGAL GUARDIANS CONSENT FORM FOR YOUNG RESEARCH PARTICIPANTS (15 to 17 years)**

**Project Title:** Neema: Formative Research on the Use of Reproductive Health Services among Youth in Kédougou and Tambacounda

IRB number: N° 00139/MSAS/DPR/CNERS du 23 Oct 2017

**Introduction:**

Hello, my name is ______________________ and I work for______________________. I am here with IntraHealth International and ideas42, two American organizations whose goal is to improve public health. We are working with the Ministry of Health and Social Action to improve reproductive health services for young people. To do this, we seek to understand the various problems they face in order to remedy them. It is important that we understand the knowledge, attitudes, and behaviors related to their health. The information we get will allow us to make recommendations.

**Participant selection:**

We are interested in speaking with people age 15-24. Are you in this age group? Are you under the age of 18? [If the participant is under 18] Would you agree for me to talk to your parent or legal guardian to allow you to participate in this interview? If so, can you introduce me to your parent or legal guardian?

***[Read to parent or legal guardian if participant is 15-17 years old]***

Your child was chosen because he or she is between 15 and 24 years old. He or she could help us understand the needs of young people in your community. We would like to know if you agree to allow your child to participates in this study. If you agree, your child will sit in private with a member of our team who will ask him or her some questions about reproductive health services. We will also ask questions about his or her experiences with these services.

We will do the interview in private and all the information that your child gives us will remain confidential. This interview will take approximately 30-40 minutes.

**Should your child participate in this study?**

Your child's participation is entirely voluntary. If you agree, you will need to sign the consent form. He / she may stop answering our questions at any time for any reason. We are talking to many other young people and all our exchanges with your child will remain confidential. We will not write down your child's name or any other personal information. An adult will be able to see us speaking with your child at all times.

**What are the disadvantages or potential benefits of participating?**

There is no direct benefit for you or your child to participate. But his/her opinion will help us to come up with recommendations to improve the health of young people. It is unlikely that there will be any risks or disadvantages in participating in this research. If he or she is uncomfortable with some of the questions, he / she may end his or her participation on the spot or move on to another question.

Regardless of your choice, no one will know whether or not you have given your consent to have your child participate.

**What if I need more information or if I have questions?**

For further information or comments on this research, please contact the Principal Investigator (details below).

**Will his/her participation in this study be kept confidential?**

All information he / she gives us will remain anonymous. His/her name will not appear on any document. All data collected will be kept confidential, the information we receive from it will be shared with other individuals for the purposes of the analysis, but in no way will we know who gave that information. We will work to remove any information that could identify your child.

**Do you have any questions?**

If you agree for your son / daughter to participate in this research, sign below. You will be given a copy of this form that you will keep.

Can we talk to him / her privately?

**Please check**

| 1. I confirm that I have received all the information and details for the participants. I have had the opportunity to ask questions and I received a full answer to those questions. |  |
| --- | --- |
| 1. I understand that my child’s participation is voluntary and he/she is free to withdraw at any time, without giving any reason. |  |
| 1. I agree for my child to participate in the above study. |  |
| 1. I agree to the recording of the interview. |  |
| 1. I agree that the research team can use anonymous quotes from this interview in the final report and in any other product of this research. |  |

**Signature** : ...........................................................Date ........................................................................

**RESEARCH PARTICIPANT CONSENT FORM (PARENTS)**

**Project Title:** Neema: Formative Research on the Use of Reproductive Health Services among Youth in Kédougou and Tambacounda

IRB number: N° 00139/MSAS/DPR/CNERS du 23 Oct 2017

**Introduction:**

Hello, my name is ______________________ and I work for______________________. I am here with IntraHealth International and ideas42, two American organizations whose goal is to improve public health. We are working with the Ministry of Health and Social Action to improve reproductive health services for young people. To do this, we seek to understand the various problems they face in order to remedy them. It is important that we understand the knowledge, attitudes, and behaviors related to their health. The information we get will allow us to make recommendations.

**Participant selection:**

We are interested in speaking with parents with children aged 10-24. Do you have a child in this age group?

**Why were you chosen?**

You were chosen because you have a child aged 10 to 24 years. However, not all parents will be able to participate in this study. If you agree to participate in this research, you can help us understand the needs of youth in your community. By agreeing to participate, you will be asked to answer questions about your knowledge and opinions about the reproductive health services available to young people, including family planning and testing for HIV and STIs.

**Should I participate?**

Your participation is entirely voluntary. If you agree, you will be asked to sign a consent form. You may stop answering questions at any time for any reason. We are talking to a lot of other people. Anything you say will remain anonymous. We will not share your name or any other personal information. This will last about 30 to 40 minutes.

**What are the possible disadvantages or advantages of participating?**

You will not benefit directly from your participation. But your opinion will help us to come up with recommendations to improve the health of young people. It is unlikely that there will be any risks or disadvantages in participating in this research. If you are uncomfortable with some questions, you can end your participation on the spot or move on to another question.

**What if I need more information or if I have questions?**

For further information or comments on this research, please contact the Principal Investigator (details below).

**Will my participation in this study be kept confidential?**

All information you give us will remain anonymous. Your name will not appear on any document. All data collected will remain confidential, the information we receive from you will be shared with other individuals for the purposes of the analysis, but in no way will we know who gave this information. We will work to remove any information that could identify you.

**Do you have any questions?**

If you agree to participate in this research, sign below. You will be given a copy of the form you will keep.

**Please check:**

| 1. I confirm that I have received all the information and details for the participants. I have had the opportunity to ask questions and I received a full answer to those questions. |  |
| --- | --- |
| 1. I understand that my participation is voluntary and I am free to withdraw at any time, without giving any reason. |  |
| 1. I agree to participate in the above study. |  |
| 1. I agree to the recording of the interview. |  |
| 1. I agree that the research team can use anonymous quotes from my interview in the final report and in any other product of this research. |  |

**Signature** : ...........................................................Date ........................................................................

**RESEARCH PARTICIPANT CONSENT FORM (HEALTH WORKERS)**

**Project Title**: Neema: Formative Research on the Use of Reproductive Health Services among Youth in Kédougou and Tambacounda

IRB number: N° 00139/MSAS/DPR/CNERS du 23 Oct 2017

**Introduction:**

Hello, my name is ______________________ and I work for______________________. I am here with IntraHealth International and ideas42, two American organizations whose goal is to improve public health. We are working with the Ministry of Health and Social Action to improve reproductive health services for young people. To do this, we seek to understand the various problems they face in order to remedy them. It is important that we understand the knowledge, attitudes, and behaviors related to their health. The information we get will allow us to make recommendations.

**Why were you chosen?**

You have been selected because you are involved in the provision of reproductive health services in a health facility. If you agree to participate in this research, you can help us understand the needs of young people in reproductive health. You will be asked to answer questions about your knowledge and opinions about the reproductive health services available to young people, including family planning and testing for HIV and STIs.

**Should I participate?**

Your participation is entirely voluntary. If you agree, you will be asked to sign a consent form. You may stop answering questions at any time for any reason. This will in no way affect your work. We are talking to a lot of other people. Anything you say will remain anonymous. We will not share your name or any other personal information. This will take 30 to 40 minutes.

**What are the disadvantages or potential benefits of participating?**

You will not benefit directly from your participation. But your opinion will help us to come up with recommendations to improve the health of young people your age. It is unlikely that there will be any risks or disadvantages in participating in this research. If you are uncomfortable with some questions, you can end your participation on the spot or move on to another question.

**What if I need more information or if I have questions?**

For further information or comments on this research, please contact the Principal Investigator (details below).

**Will my participation in this study be kept confidential?**

All information you give us will remain anonymous. Your name will not appear on any document. All data collected will remain confidential, the information we receive from you will be shared with other individuals for the purposes of the analysis, but in no way will we know who gave this information. We will work to remove any information that could identify you.

**Do you have any questions?**

If you agree to participate in this research, sign below. You will be given a copy of the form you will keep.

**Please check:**

| 1. I confirm that I have received all the information and details for the participants. I have had the opportunity to ask questions and I received a full answer to those questions. |  |
| --- | --- |
| 1. I understand that my participation is voluntary and I am free to withdraw at any time, without giving any reason. |  |
| 1. I agree to participate in the above study. |  |
| 1. I agree to the recording of the interview. |  |
| 1. I agree that the research team can use anonymous quotes from my interview in the final report and in any other product of this research. |  |

**Signature** : ...........................................................Date ........................................................................
